# Using mechanistic models for the clinical interpretation of complex genomic variation

**DOI:** 10.1038/s41598-019-55454-7

**Published:** 2019-12-12

**Authors:** María Peña-Chilet, Marina Esteban-Medina, Matias M. Falco, Kinza Rian, Marta R. Hidalgo, Carlos Loucera, Joaquín Dopazo

**Affiliations:** 1 Clinical Bioinformatics Area. Fundación Progreso y Salud (FPS). CDCA, Hospital Virgen del Rocío, 41013 Sevilla, Spain; 2 Bioinformatics in RareDiseases (BiER). Centro de Investigación Biomédica en Red de Enfermedades Raras (CIBERER), FPS, Hospital Virgen del Rocío, 41013 Sevilla, Spain; 3 Bioinformatics and Biostatistics Unit, Centro de Investigación Príncipe Felipe (CIPF), 46012 Valencia, Spain; 4 INB-ELIXIR-es, FPS, Hospital Virgen del Rocío, Sevilla, 42013 Spain

**Keywords:** Cellular signalling networks, Systems analysis

## Abstract

The sustained generation of genomic data in the last decade has increased the knowledge on the causal mutations of a large number of diseases, especially for highly penetrant Mendelian diseases, typically caused by a unique or a few genes. However, the discovery of causal genes in complex diseases has been far less successful. Many complex diseases are actually a consequence of the failure of complex biological modules, composed by interrelated proteins, which can happen in many different ways, which conferring a multigenic nature to the condition that can hardly be attributed to one or a few genes. We present a mechanistic model, *Hipathia*, implemented in a web server that allows estimating the effect that mutations, or changes in the expression of genes, have over the whole system of human signaling and the corresponding functional consequences. We show several use cases where we demonstrate how different the ultimate impact of mutations with similar loss-of-function potential can be and how the potential pathological role of a damaged gene can be inferred within the context of a signaling network. The use of systems biology-based approaches, such as mechanistic models, allows estimating the potential impact of loss-of-function mutations occurring in proteins that are part of complex biological interaction networks, such as signaling pathways. This holistic approach provides an elegant alternative to gene-centric approaches that can open new avenues in the interpretation of the genomic variability in complex diseases.

## Introduction

The extraordinarily fast increase in throughput of sequencing technologies in the last decade^1,2^ has fostered different international collaborative projects^3–5^ that resulted in an unprecedented increase in our knowledge of the mutational spectrum of diseases, especially thosewith significant morbidity and mortality and caused by highly penetrant (typically protein-coding) variants^6,7^. However, in addition to the expected pathogenic variation, these projects have revealed an unanticipated amount of variation at genome level in apparently normal, healthy individuals. Actually, putative loss-of-function (pLoF) variants, with a potential severe effect on the function of human protein-coding genes^8^ seems to be surprisingly pervasive, according to reports from different genome sequencing projects^3,4,9^. Conservative estimates suggest that there are more than 250 pLoF variants predicted to be highly damaging^10^ per sequenced genome, in protein coding regions^8^, as well as other non-coding regions, such as miRNAs^11^, transcription factor binding sites^12^ and others^13^. Therefore, a better understanding on the contribution of pLoF to disease is critical for clinical applications of genomic data^14^.

From a historical perspective, the application of sequential heuristic filters, in a process called prioritization, has demonstrated to be a useful tool for the clinical interpretation of genomic variation in rare Mendelian diseases^6,7^. Thus, extensively used filtering criteria are: (i) the potential impact of the variant in the resulting gene product, estimated by different indexes that predict the potential pathologic effect of an amino acid substitution (e.g. Polyphen^15^, SIFT^16^, SNPeffect^17^, PMUT^18^, etc.) that can be combined with allelic frequency, conservation, etc. (e.g. PROVEAN^19^, PupaSNP^20^, CONDEL^21^, VAAST^22^, MutationTaster^23^, etc.); (ii) variant population frequencies, given that variants with a relatively high frequency in the population are unlikely to be causative of many hereditary disorders (obtained from different repositories such as the 1000 genomes^3^, the Exome Aggregation Consortium^24^, the gnomAD^25^, or also from local population repositories, which have demonstrated to be useful for this purpose^26^); (iii) evolutionary conservation (e.g. PhyloP^27^, GERP^28^, etc.); (iv) compendiums of different criteria, such as CADD^29^ or, more recently, based on artificial intelligence^30^. These filters can also be used in combination with knowledge on functional labels^31^, syndromes and phenotypes^32^, or diseases^33–35^, previously associated to the most likely candidate genes, as implemented in tools such as Phen-Gen^36^, eXtasy^37^, PhenIX^38^, Exomiser^39^, etc. Different computer applications, such as Annovar^40^, the Variant Effect Predictor^41^ or the CellBase^42^, collect all this information that is subsequently used by different web interfaces that allow carry out this prioritization interactively, likeOVA^43^, BiERapp^44^, QueryOR^45^ or Mutation Distiller^46^, etc.

Despite success of gene centric clinical interpretation of variation in finding disease genes in single gene Mendelian disorders^7,47,48^, the application of these concepts to complex diseases has produced more modest results^49^. Contrarily to the case of RDs, complex diseases are characterized by phenotypic heterogeneity, that is, patients with similar presentations often have different underlying disease mechanisms, and incomplete penetrance, as a consequence of its multigenic nature and the significant role of the environment^50–52^. However, the most widely used tools for the interpretation of genetic variation all focus on monogenic genetic models or, on oligogenic ones as much.

As a matter of fact, complex, multigenic diseases can be better understood as failures of functional modules caused by different combinations of perturbed gene activities rather than by the failure of a unique gene^53^. Actually, the idea of cell functionality as a result of the complex interactions between their molecular components is not new^54^ and was proposed almost two decades ago in the context of systems biology^55^. These interacting components define operational entities or modules to which different elementary functions can be attributed. This modularity, extensively described in numerous reports^56,57^, suggests that causative genes for the same disease often reside in the same biological module, which can be a protein complex or any type of biological network^58,59^. Currently, a detailed recapitulation of the knowledge on biological networks that account for cell functionality, metabolism and other cell processes is available in different pathway repositories such as the Kyoto Encyclopedia of Genes and Genomes (KEGG)^60^, Reactome^61^, Pathway Commons^62^, Wikipathways^63^ and others, including pathways with specific, curated descriptions of disease mechanisms^64^. Since pathways describe how proteins interact among them to trigger cell functionalities or to generate different molecules and metabolites, mathematical models of the activity of such pathways constitute ultimately mechanistic descriptors of the behavior of the cell. In fact, recent reports demonstrate that mechanistic models of the activity of metabolic or signaling pathways, render highly precise predictions of complex phenotypes, such as patient survival^65,66^, drug response^67^, etc.

An interesting property of mechanistic models is that, in addition to study molecular mechanisms of disease in a particular condition, they can be used to predict the potential consequences that perturbations (mutations or changes in the expression) of the proteins that compose the pathway can have over the individual circuits that trigger cell actions or the production of metabolites^68,69^. This mechanistic view of the effects of a change in the integrity or the activity of one or several proteins within the context of signaling^66^ or metabolic^70^ pathways can be used to understand the functional consequences of pLoF mutations and/or gene expression perturbations, thus providing a clinical interpretation for these variations in complex scenarios that takes into account the whole context of the impact of the perturbation from a functional angle.

Here we present a web server that implements a version of the *Hipathia*^66^, an algorithm that has recently been shown that outperforms other current competing algorithms^71^, and demonstrate the advantages of mechanistic models in the interpretation of complex variability in two scenarios of different complexity: a rare disease, Fanconi anemia (FA) and a common disease, diabetes.

## Implementation

### Overview

The general idea for the interpretation of complex variability is based on the use of an algorithm that uses gene expression (as a proxy of the presence of the corresponding protein within a pathway) to model the activity of signaling circuits defined within signaling pathways, which ultimately provide a hint on functional cell activity. A functional assessment of the differences in functional cell behavior between two conditions can be achieved by comparing the corresponding signaling circuit activity profiles to detect what circuits behave differently. Since the model only requires gene expression and the topology of the signaling circuits, it is easy to simulate a “mutated” condition just by reducing or setting to zero the expression of one or several genes (in the assumption that the effect in signal transduction of an inactive protein is equivalent to its absence) and constructing an artificial gene expression profile for the simulated mutated condition. Then, the original condition can be compared to the simulated condition to detect the circuits and the corresponding functions affected by the LoF of the genes.

**Figure 1:**
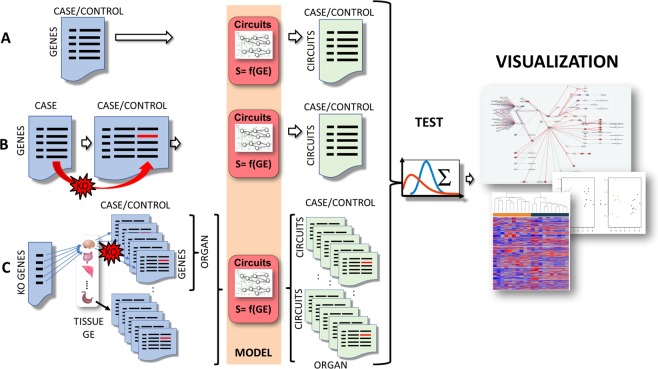
Schematic representation of the three analysis scenarios implemented in the *Hipathia* web server. (**A**) Conventional transcriptomics case/control study transformed by *Hipathia* into a differential signaling circuit activity contrast. (**B**) Interactive simulation of the effect of a mutation over the signaling circuits. (**C**) Estimation of the potential effect of a list of mutations over the different signaling circuits in a number of selected human tissues. Icons for organs were taken from the Reactome Icon Library^111^

Figure 1 shows the schema of the three analysis scenarios implemented in the *Hipathia* web server. The simplest scenario, Differential Signaling, (Fig. 1A) represents a conventional transcriptomics case/control study that can be transformed into a differential signaling circuit activity contrast. Gene expression values are used to define circuit activities (see below for details) and a Wilcoxon test is used to detect circuits that are differentially activated between cases and controls. In the second scenario, the Perturbation effect, the effect of a LoF mutation is interactively simulated in one or several genes (Fig. 1B). To carry out this simulation the gene expression corresponding to a specific condition is uploaded. The graphical interface allows the user to select one or several genes and decide the mutation effect by modulating the expression value of the selected genes (setting the value to 0 would simulate a total loss of function mutation and raising the value would simulate an over expression). Then, a fold change between the original and the simulated conditions is used to detect effects of the perturbation over the different circuits. The third scenario, Variant interpreter, (Fig. 1C) allows estimating the potential effect of a list of mutations over different human tissues. To achieve so, the GTEx gene expression matrices corresponding to 30 different tissues (see Additional Table 1), containing each a variable number of individuals, are used as controls and then, equivalent matrices of cases are generated by simulating the mutations as previously described on all the individuals. Then a case/control contrast with a Wilcoxon test is carried out for each tissue, which would reveal whether some of the mutations in the list have a significant impact on one or several tissues and the functional nature of such impact.

### The web interface

The input of the program consists of normalized gene expression matrices in CSV format for the two first options of Differential signaling activity and the Perturbation effect (Fig. 1A,B) and also optionally for the Variant interpreter option that explores the effect of mutations across tissues (Fig. 1C), as user defined tissue. Expression may have been measured with any sequencing or microarray technology. The gene expression matrix must include samples as columns and genes as rows. Gene names must be Entrez or HUGO IDs.

For the Variant Interpreter option, a list of Entrez or HUGO gene names can be provided.

### Graphical representation of the results

Different analysis types are carried out on the circuit’s activities calculated, which include two-class comparisons and PCA, with the corresponding visualizations as heatmaps and PCA plots. Graphical representation of the circuits significantly up- or down-activated, including the individual node expression change, are also provided (see Fig. 1 right). An interactive graphical output in which the pathways analyzed are displayed with the possible ways in which the signal can be transmitted from receptor proteins to the corresponding effector proteins, highlighting those in which significant changes in signaling are found. In this visual representation, disruptions or activations in the signal transduction caused by gene perturbations (mutations or expression changes) can be easily visualized and understood in terms of their consequences on cell signaling and their ultimate effect over the corresponding functions triggered by the effectors.

The client of the web application has been implemented in JavaScript using the HTML5 and SVG standards and uses CellMaps^72^ libraries for interactive visual representation of pathways.

### Mechanistic model of cell functionality triggered by signaling

The *Hipathia* (acronym for High-throughput pathway interpretation and analysis) is a mechanistic model of signaling circuit activities previously described^66^. In brief, circuits that connect receptor proteins to specific effector proteins, which ultimately trigger cell activities, are defined using KEGG pathways^60^. Such circuits represent the sequence of activation (and inhibition) steps that mediates the transduction of the signal from the receptor to the effector protein. The method assumptions are that, in order to transduce the signal, all the proteins that connect the receptor with the effector should be present and the higher the amount of these proteins the stronger will be the signal. Measurements of mRNA levels are taken as proxies of the amount of the corresponding proteins (a quite common assumption^73–78^). Then, in order to quantify the intensity of signal transduction, the following steps are taken: normalized gene expression values, rescaled to a value in the range [0,1], obtained as explained above, are used as proxies of the protein activities (activations or inhibitions in the transmission chain)^73,75,79^. Thus, the intensity value of signal transduced along a circuit that reaches the effector is estimated by starting with initial signal intensity with the maximum value of 1 in the receptor, which is propagated along the nodes of the signaling circuits according the recursive formula:

1 $${S}_{n}={\upsilon }_{n}\cdot (1-\prod _{{s}_{a}\in A}(1-{s}_{a}))\cdot \prod _{{s}_{i}\in I}(1-{s}_{i})$$

where *S*_*n*_ is the signal intensity for the current node *n*, *v*_*n*_ is its normalized gene expression value, *A* is the set of activation signals (*s*_*a*_), arriving to the node *n* from the corresponding activation edges, *I* is the set of inhibitory signals (*s*_*i*_) arriving to the node from inhibition edges^66^. Like normalized gene expression values, circuit activity values are measurements with no absolute meaning by themselves but rather in a comparison.

The application of this formula to all the circuits defined in all the pathways allows transforming a gene expression profile into the corresponding signaling circuit activity profile for any sample studied. If two conditions are compared, a Wilcoxon test can used to assess differences in signaling circuit activity between both types of samples.

### Estimation of the impact of a mutation over cell functionality

The effect of a mutation is dependent on the context which includes the activity (gene expression status) and the integrity (mutational status) of the rest of proteins involved in the pathways that trigger functionalities relevant to the disease analyzed (disease hallmarks). The effect of one or several simultaneous mutations in a specific tissue can easily be predicted using the mechanistic model^68,69^. The reference or control dataset is taken from the tissue of interest in GTEx^80^. Then, an affected dataset is simulated from the control dataset by drastically reducing the expression of the gene(s) with a pLoF mutation by multiplying their expression values by 0.01 in all the control samples. This simulates either an inactive gene or a non-functional gene product. Then, the circuit activities are recalculated in the affected dataset and it is compared to the reference dataset. Although not completely realistic, given that the model does not have information on the way in which the diseased tissue will transcriptionally react to the perturbation induced by the mutated genes, the results will certainly point with precision to those cell functions affected in first instance.

### Data Sources

In the current version of *HiPathia* more than 8000 circuits have been identified and modeled within a total of more than 150 pathways downloaded from KEGG^60^ corresponding to three species (human 145, mouse 141 and rat 141).

Gene expression data from 30 non-diseased tissue sites (See Additional Table 1) used in the third option were taken from the GTEx Portal^80^ (GTEx Analysis V7; dbGaP Accession phs000424.v7.p2).

### Data and methods for the examples

Gene expression for bone marrow, which is not present in GTEx, was downloaded from the Gene Expression Omnibus (GEO) database (GSE16334)^81^.

Gene expression microarray study that compares human islets gene expression from 54 non-diabetic and 9 type 2 diabetic donors^82^ was downloaded from GEO (GSE38642).

Data on natural variability of different populations, which comprises over 88 million variants of 2,504 individuals from 26 populations, was obtained from the 1000 Genomes project portal^3,83^.

In order to assess the impact of the natural variation found in genes of healthy population, variants located within gene regions were annotated using CADD^29^. As proposed by CADD developers, a gene was considered to carry a pLoF mutation when the CADD score is over the threshold of 20^84^. A gene is considered to be affected by pLoF in a recessive scenario, when the two alternative alleles are present.

### Transcriptomics data processing

Gene expression data from microarrays were summarized and normalized by quantiles with the Robust Multiarray Analysis method using *affy* R package^85^. Probes were mapped to the corresponding genes using BiomaRt^86^. Gene expression values are estimated as the 90 percentile of probe expression values. Probes that mapped in more than one gene were discarded (except in the case that they were the unique probes mapping on the gene, that the median value of intensities was taken.)

RNA-seq gene expression data were normalized with the Trimmed mean of M values (TMM) normalization method using the *edgeR* package^87^.

Then, the *Hipathia*^66^ algorithm requires some extra steps for the calculation of the signal intensities. Thus, a logarithm transformation (apply log(matrix + 1)) followed by a truncation by the quantile 0.99 (all values greater than quantile 0.99 are truncated to this upper value, all values lower than quantile 0.01 are truncated to this lower value) were applied to the normalized gene expression values. Finally, in both cases, quantiles normalization using the *preprocessCore*R package^88^ was carried out.

## Results

We demonstrate the possibilities that mechanistic models offer for the interpretation of genomic variability in two different scenarios. The first one is the case of Fanconi Anemia (ORPHA:84), a rare disease with well-known hallmarks (chromosomal instability caused by failures in the repair machinery) which have been mapped in the FA pathway. Therefore, variants having an impact in the pathway, in the absence of other considerations, are potential genetic disease drivers. However, if they affect a large number of pathways as well, then they are probably lethal genes rather than disease driver genes.

A second scenario considered a complex disease, such as type 2 diabetes, where their disease hallmarks are not so well defined. Some general cell processes could potentially be associated with the disease phenotype but, in many cases, these are too general to be considered a clear causative agent of diabetes. Then, the number of cell processes potentially related to the disease can be reduced by considering only those that display a significantly differential behavior in a case-control comparison.

### Fanconi anemia, a case example of a rare disease

**Figure 2:**
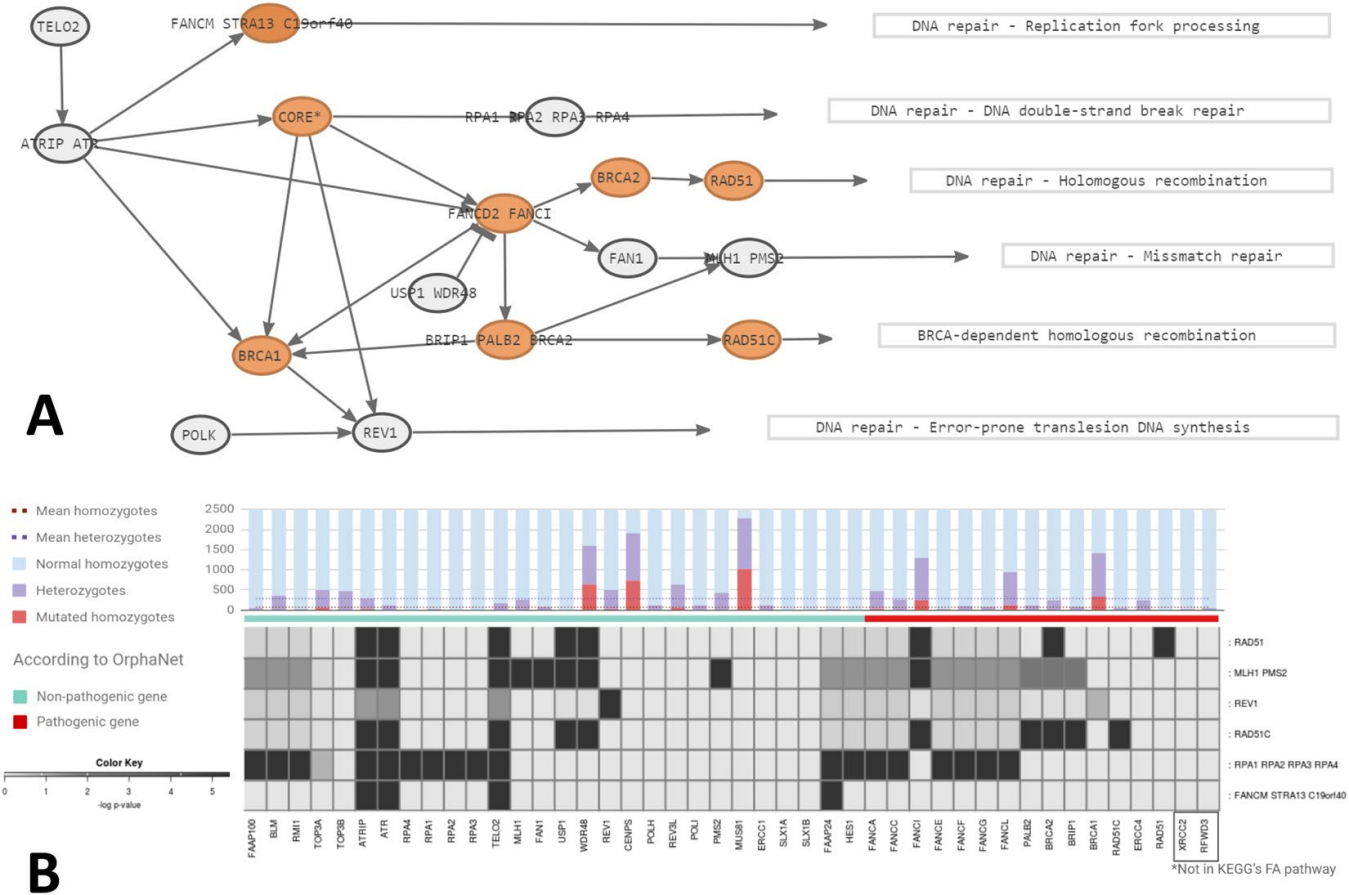
Fanconi anemia simulation. (**A**) The FA pathway. Nodes in orange contain known FA pathogenic genes. (**B**) The result of the LoF simulations over the different FA circuits. Over each gene column, the frequencies at which these genes have been found in the normal population (1000 genomes) with a pLoF mutation is represented.

A detailed map of FA signaling is available in KEGG (ID: 03460) and is already implemented in the corresponding mechanistic model in *Hipathia*. Figure 2A shows the basic map of the signal transduction chain used to relate gene expression to the activity of signaling circuits within the FA pathway, which ultimately trigger cell activities related to FA hallmarks.

Since the main tissue affected by FA, bone marrow, is not present in GTEx, we uploaded to *Hipathia* data from an experiment available in the GEO database (GSE16334) in which bone marrow from healthy donors was analyzed^81^.

The Loss of function (LoF) mutations in all the genes in the pathway was simulated and the results are shown in Fig. 2B. As expected, known disease genes produced more impact over the FA pathway than most of the genes that have not been described as disease-causing (to date). Other genes not identified as causal genetic drivers of FA produce a devastating effect on the FA circuits, but also affect many other signaling circuits in other pathways (see Additional Table 2), and the LoF mutations in them were almost absent in the healthy population (data taken from 1000 genomes), which suggest that these genes are lethal and, consequently, unrelated with the disease.

### Diabetes as a common disease case example

Diabetes is a highly prevalent disease, affecting to more than 8% of the population and being a major cause of blindness, kidney failure, heart attacks, stroke and lower limb amputation^89^. Inflammation is one of the most prominent phenotypic consequences of diabetes^90^. However, inflammation is a too general concept that can be associated to many conditions and, consequently, focusing on all the circuits that directly or indirectly trigger inflammation could result in the detection of activations unrelated to the disease. Therefore, a diabetes gene expression dataset that compares human islets gene expression of healthy donors with type 2 diabetic patients^82^ was used to determine which, among the 30 inflammation-related circuits, were specifically deregulated in the disease. Additional Table 3 shows three inflammation-related circuits significantly deregulated in diabetic human islets with respect their normal counterparts (FDR-adjusted p-value < 0.05)^91^, which are *Rap1 signaling pathway:PRKCI-PARD6A-PARD3, NF-kappa B signaling pathway: CCL19* and *NF-kappa B signaling pathway: CCL21*.

**Figure 3:**
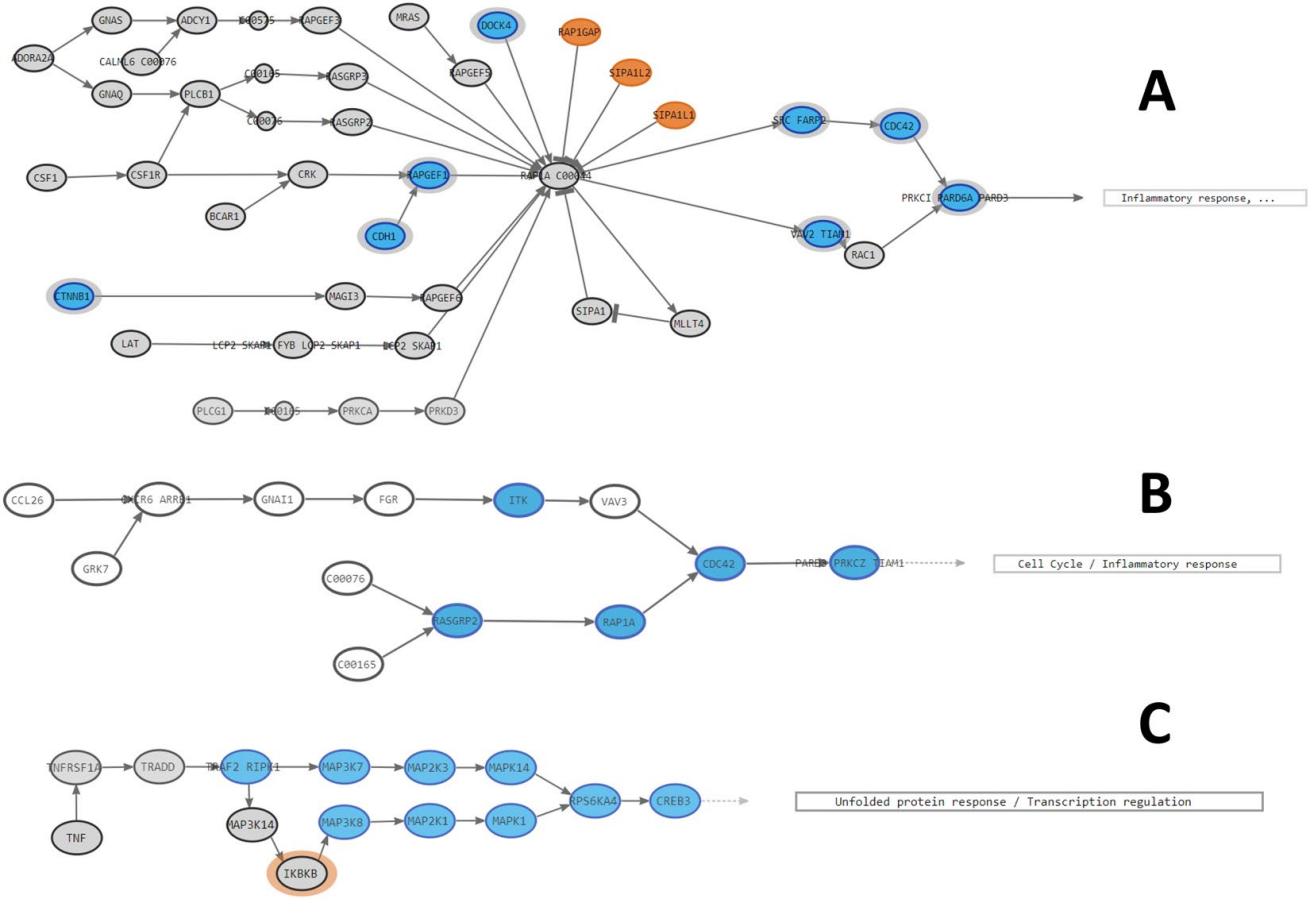
Signaling circuits analyzed in the diabetes case study. Nodes in orange contain genes whose LoF causes an upregulation of the circuit and nodes in blue contain genes whose inactivation causes circuit downregulation. (**A**) *Rap1 signaling pathway:PRKCI PARD6A PARD3*, (**B**) *Chemokynes pathway:PARD3-PRKCZ-TIAM1* and (**C**) *TNF signaling pathway:CREB3* circuits.

In order to demonstrate the suitability of mechanistic models for the interpretation of complex genomic variation we have focused on three circuits that represent three different scenarios. Firstly, the circuit *Rap1 signaling pathway:PRKCI-PARD6A-PARD3* (Fig. 3A) that triggers inflammation and displays one of the highest differences in activity between diabetic and non-diabetic samples. Secondly, the signaling circuit *Chemokynes pathway:PARD3-PRKCZ-TIAM1* (Fig. 3B), which also triggers inflammation but does not display any significant difference in the comparison (see Additional Table 3), which would represent a circuit with a disease hallmark but unrelated with the disease. And finally, the signaling circuit *TNF signaling pathway:CREB3* (Fig. 3C), which neither presents a different activity in the comparison nor the functions triggered are likely to be directly related with diabetes (see Additional Table 4).

**Figure 4:**
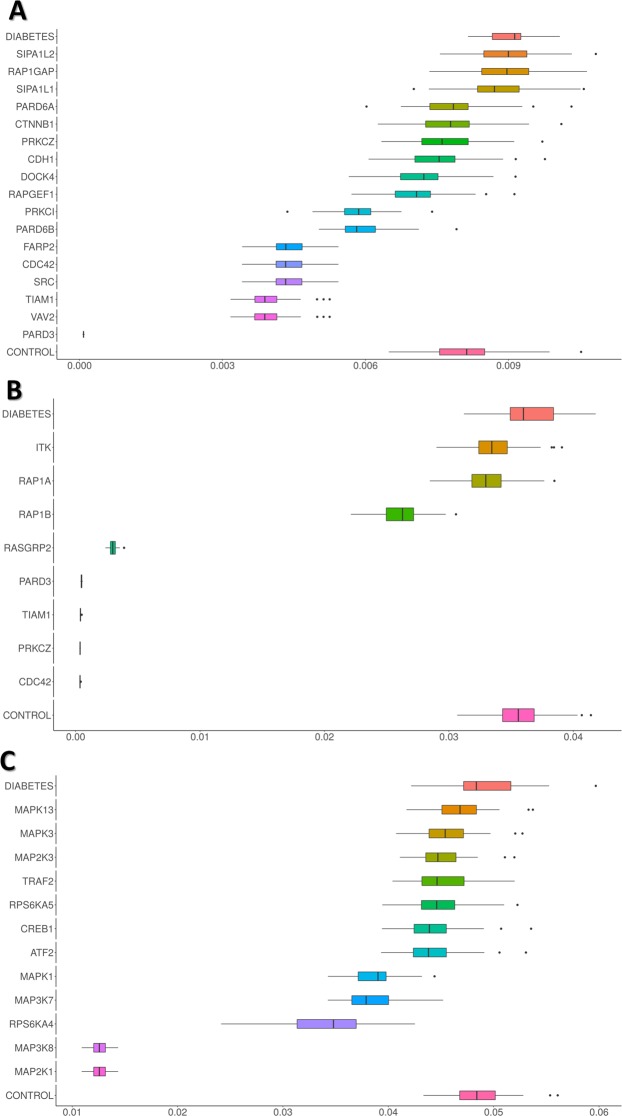
Effect of genes with LoF mutations using the pancreatic islet tissue as used defined tissue, and comparing the resulting pathway activity after the simulations with those displayed by both the normal and the type 2 diabetes tissues. (**A**) *Rap1 signaling pathway:PRKCI PARD6A PARD3*, (**B**) *Chemokynes pathway:PARD3-PRKCZ-TIAM1* and (**C**) *TNF signaling pathway:CREB3* circuits.

Then we used the *Hipathia* functionality to check the effect of genes with LoF mutations using the pancreatic islet tissue as user-defined tissue, and comparing the results of the resulting pathway activity after the simulations with those displayed by both the normal and the type 2 diabetes tissues. Figure 4 shows the results on the LoF simulations in the three above mentioned signaling circuits. Three genes, *SIPA1L2*, *RAP1GAP* and *SIPA1L1*, produce in the *Rap1 signaling pathway:PRKCI-PARD6A-PARD3* circuit a level of activity in the control quite similar to the observed in the diabetic tissue. However, neither in the *Chemokynes pathway:PARD3-PRKCZ-TIAM1* nor in the *TNF signaling pathway:CREB3* circuits this trend is observed.

Interestingly, when the differential activity caused by simulating LoF mutations in the three circuits is compared with the frequency at which these genes appear in the healthy population with a homozygous LoF mutation, it is clear that genes with relatively high LoF variation frequencies cause no effect in the pathway activity as a general rule (see Additional Fig. 1).

## Discussion

Mechanistic models of pathway activity can be considered (complete or partial) representations of disease mechanisms and have been successfully used to uncover details on the molecular mechanisms behind different cancers^65,66,92–94^, common diseases^95,96^, rare diseases^97^ or the mechanisms of action of drugs^67,98^. Moreover, mechanistic models have been used in other biologically interesting scenarios such as the discovery of molecular mechanisms that explain how stress-induced activation of brown adipose tissue prevents obesity^99^ or the mechanisms of death and the post-mortem ischemia of a tissue^100^. Therefore, mechanistic models provide a holistic and accurate framework for the interpretation of the consequences of the genetic variation found in whole exome or genome sequencing, especially in the case of complex diseases, where the effect can be highly dependent on the specific condition of the patient.

However, even in rare Mendelian diseases, typically caused by one or a few highly penetrant variants^6,7^, the scenario is sometimes far away from the expected simplicity, as the example with FA reveals. When the LoF mutations were simulated, *WDR48* LoF displayed a surprisingly high impact over *RAD51*, *MLH1/PMS2* and *RAD51C* circuits. However, a large number of healthy individuals carry potential deleterious mutations in this gene, as shown in Fig. 2. Protein *WDR48* acts as a complex together with *USP1* to inhibit *FANCD2/FANCI* complex (Fig. 2A), indeed the coupling of *WDR48* to the distal end of *USP1* dramatically enhances *USP1* activity, which catalyzes the deubiquitination of *FANCD2*. Thus, is *USP1* that plays the catalytic role, whilst *WDR48* is only required to join *USP1*^101,102^. Therefore, *WDR48* gene may accumulate mutations that do not affect WD repeat domain structure, and that maintain its union with *USP1*intact, mutations that would be predicted as deleterious in other genes^103^.

A similar situation was observed with *FANCI*, that also showed an impact over *RAD51*, *MLH1/PMS2* and *RAD51C* signaling circuits, but we found several individuals harboring homozygous mutations in this gene. Protein complex *FANCI/FANCD2* is required to DNA repair function^104^, however, *FANCI* appears to be more naturally mutated in normal population than *FANCD2* (Additional Table 2, data on LoF simulation are not available since *FANCD2* expression was not present in the analyzed dataset), suggesting that *FANCD2* may play a more determinant role than *FANCI* in the DNA repair-related function of the complex.

LoF simulations of *ATRIP*, *ATR* and *TELO2* have an important impact over FA pathway, given that affects 5 out of 6 sub-pathways. Moreover, all three genes have a low frequency of mutations in 1000 genomes healthy population, which point to them as good candidates. However, none of these genes are categorized as pathogenic by Orphanet. Nevertheless, *ATR* LoF simulation affects not only FA pathway, but also to 38 more circuits, most of them belonging to p53 signaling pathway. This fact suggests that ATRIP/ATR complex is relevant to many other cellular processes, so, while detected as good candidates with high impact over the cell, its impact might not be specific of FA disease. Protein *TELO2* functions as an S-phase checkpoint protein in the cell cycle, aside of its role in DNA repair, indeed mutations in this gene result in severe developmental diseases. Therefore, it is not misguided to present this gene as a candidate gene to further study.

Interestingly, only 12.29% of LoF mutations categorized as pathogenic by Orphanet have no impact over FA pathway, while 35.71% of the non-pathogenic genes have no impact on the pathway according to our model (Additional Table 2). This suggests that our model can be a useful tool to provide variant interpretation, especially in those cases where no variant is found in the disease-associated known genes.

As for Rap1 signaling pathway:PRKCI-PARD6A-PARD3 LoF simulation analysis related to diabetes, we found up to nodes disturbing the activation of the sub-pathway: CTNNB1, CDH1, RAPGEF1, DOCK4, RAP1GAP, SIPA1L2, SIPA1L1, SRC/FARP2, CDC42, VAV2/TIAM1 and PKKCI/PARD6A/PARD3.Among them, the LoF of nodes RAP1GAP, SIPA1L2 and SIPA1L1 were the only ones showing diabetes-like activation values, therefore resulting in an overactivation of the Rap1 signaling pathway:PRKCI-PARD6A-PARD3 circuit, as found in the analysis performed with the diabetes dataset. As seen in Fig. 3A, all three genes code for proteins that inactivate the RAS-related RAP1 protein, which is involved in many cellular processes, including inflammatory response, cell proliferation and adhesion and Thyroid Stimulating Hormone (TSH) signaling pathway.

The relationship between inflammation and type 2 diabetes has long been established. The proposed mechanisms to explain impaired insulin secretion include oxidative stress, endoplasmic reticulum stress, ectopic lipid deposition in muscle, liver and pancreas, lipotoxicity and glucotoxicity, all processes that may cause an inflammatory response^105,106^.

Focusing on *RAP1* inactivation, a recent study reported the association between inhibition of *RAP1* (via miRNAs) and hyperglycemia in patients with Type 1 Diabetes^107^. Moreover, other authors have suggested its relation with diabetic cardiomyopathy and with immune-mediated diabetes^108,109^. Therefore, it is reasonable to think that LoF mutations in those genes inactivating RAP1 protein would result in an over-activation of inflammatory response and thus, an increased risk of developing immune-mediated diabetes.

Beyond signaling, which can be mechanistically linked to cell functionalities^71^, other cellular processes such as metabolism, can also be used for a detailed interpretation of the consequences of variation over other relevant cell biological processes. Actually, it has recently been demonstrated that mutations with an impact over several metabolic pathways have a clear connection with certain cancer processes and could be easily related to patient phenotypes such as survival^70,92^.

Mechanistic models like the one used in this work rely on the accuracy of the pathways used to derive circuit topologies^71^. Although in general pathways from the most known repositories (KEGG^60^, Reactome^61^, Pathway Commons^62^, Wikipathways^63^, etc.) represent curated biological knowledge and are expected to be quite accurate, they are not absent of errors. Moreover, in many cases pathways represent incomplete biological knowledge. Errors or missing links will define erroneous or incomplete circuits with unpredictable (but most likely erroneous) behavior. Recently, we have described some constitutively inactive circuits that were incorrectly annotated in the Fanconi Anemia pathway that, after literature exploration, could be fixed and recovered the expected behaviour^110^.

## Conclusions

By transforming the current variant-centric and gene-centric data into function-centric measurements a more holistic and context-dependent approach to the interpretation of complex variability, especially useful for common diseases, can be attained. Mechanistic models of cell functionality will open new avenues for understanding the complex relationships among genes that ultimately shape the phenotype.

### Availability and requirements

Project name: HiPathia. Project home page: http://hipathia.babelomics.org. Operating system(s): Platform independent. Programming language: HTML/JavaScript in the frontend; Java, R and web services in Node and Express for the backend. Other requirements: Modern browsers. License: Does not apply. Any restrictions to use by non-academics: no

## Supplementary information

Supplementary information 

## Data Availability

The datasets analyzed during the current study are available in the GEO repository (accession: GSE16334) [https://www.ncbi.nlm.nih.gov/geo/], GTEx portal (dbGaP accession phs000424.v7.p2) [https://www.gtexportal.org/home/].
