# Supplementary material for: Using mechanistic models for the clinical interpretation of complex genomic variation
Source: Sci Rep. 2019 Dec 12;9:18937. doi: 10.1038/s41598-019-55454-7 (PMC6908734; doi:10.1038/s41598-019-55454-7)

Using mechanistic models for the clinical interpretation of complex genomic variation

María Peña-Chilet, Marina Esteban-Medina, Matias M. Falco, Kinza Rian, Marta R. Hidalgo, Carlos Loucera, Joaquín Dopazo

Additional Table 1.Gene expression values of 30 non-diseased tissues. The 30 non-diseased tissue sites used in the current version of Hipathia, taken from the GTEx Portal

Tissue name	Samples
**Adipose Tissue**	797
**Adrenal Gland**	190
**Bladder**	11
**Blood**	537
**Blood Vessel**	913
**Brain**	1671
**Breast**	290
**Cervix Uteri**	11
**Colon**	507
**Esophagus**	1021
**Fallopian Tube**	7
**Heart**	600
**Kidney**	45
**Liver**	175
**Lung**	427
**Muscle**	564
**Nerve**	414
**Ovary**	133
**Pancreas**	248
**Pituitary**	183
**Prostate**	152
**Salivary Gland**	97
**Skin**	1203
**Small Intestine**	137
**Spleen**	162
**Stomach**	262
**Testis**	259
**Thyroid**	446
**Uterus**	111
**Vagina**	115

Additional Table 2. Effect of LoF mutations in the genes within the FA pathway over the activity of signaling circuits. The table contains the effect of a LoF mutations in the genes comprising the FA pathway and the corresponding effect on circuits of the FA pathways and circuits of other pathways, along with the occurrence of this type of mutations in the healthy population (1000 genomes)

Gene Symbol	FA-association	pLI	% homozygotes	Total circuits affected	FA circuits affected	p-value
**FAAP100**	NP	0.08826	0.039936102	1	1	0.00529
**BLM**	NP	4.139E-11	0.519169329	1	1	0.00529
**RMI1**	NP	0.003097	0.039936102	1	1	0.00529
**RMI2**	NP	0.0246	13.21884984	NA	NA	NA
**TOP3A**	NP	2.636E-08	2.076677316	0	0	NA
**TOP3B**	NP	0.1145	0.039936102	0	0	NA
**ATRIP**	NP	0.001487	0.878594249	5	5	0.00106
**ATR**	NP	0.7013	0.159744409	38	5	0.00016
**RPA4**	NP	0.274	0	1	1	0.00529
**RPA1**	NP	0.3087	0	1	1	0.00529
**RPA2**	NP	0.8885	0	1	1	0.00529
**RPA3**	NP	0.00007563	0	1	1	0.00529
**TELO2**	NP	4.842E-07	0.159744409	5	5	0.00106
**MLH1**	NP	0.7396	0.439297125	1	1	0.00529
**FAN1**	NP	1.468E-15	0	1	1	0.00529
**USP1**	NP	0.9932	0.039936102	3	3	0.00176
**WDR48**	NP	0.9998	25.79872204	3	3	0.00176
**REV1**	NP	0.997	1.277955272	1	1	0.00529
**CENPX**	NP	0.592	0	NA	NA	NA
**CENPS**	NP	0.008521	29.23322684	0	0	NA
**POLH**	NP	0.003591	0	0	0	NA
**POLK**	NP	5.999E-07	0	NA	NA	NA
**REV3L**	NP	1	1.837060703	0	0	NA
**POLI**	NP	4.27E-09	0.039936102	0	0	NA
**PMS2**	NP	1.139E-13	0.559105431	1	1	0.00529
**MUS81**	NP	0.00000157	41.014377	0	0	NA
**EME1**	NP	7.067E-11	0.638977636	NA	NA	NA
**EME2**	NP	9.501E-14	0	NA	NA	NA
**ERCC1**	NP	0.0002869	0	0	0	NA
**SLX1A**	NP	0.6255	0.079872205	0	0	NA
**SLX1B**	NP	0.5168	0	0	0	NA
**FAAP24**	NP	0.00002662	0	2	2	0.00265
**HES1**	NP	0.6441	0	5	1	0.00106
**POLN**	NP	2.997E-32	13.77795527	NA	NA	NA
**FANCM**	P	6.245E-12	0.039936102	NA	NA	NA
**FANCA**	P	2.081E-34	1.517571885	1	1	0.00529
**FANCB**	P	0.9884	0	NA	NA	NA
**FANCC**	P	5.88E-10	0.279552716	1	1	0.00529
**FANCD2**	P	2.479E-14	0.479233227	NA	NA	NA
**FANCI**	P	5.165E-19	9.265175719	3	3	0.00176
**FANCE**	P	0.003139	0	1	1	0.00529

**FANCF**	P	0.003815	0.079872205	1	1	0.00529
**FANCG**	P	9.753E-09	0	1	1	0.00529
**FANCL**	P	0.00000002	4.552715655	1	1	0.00529
**PALB2**	P	1.326E-10	0	1	1	0.00529
**BRCA2**	P	2.213E-15	0.079872205	2	2	0.00265
**BRIP1**	P	5.378E-13	0	1	1	0.00529
**BRCA1**	P	3.287E-20	13.57827476	0	0	NA
**RAD51C**	P	4.278E-09	0	1	1	0.00529
**UBE2T**	P	0.0003692	0	NA	NA	NA
**ERCC4**	P	3.332E-10	0.239616613	0	0	NA
**SLX4**	P	0.00000008	1.277955272	NA	NA	NA
**RAD51**	P	0.9904	0	1	1	0.00529
**MAD2L2**	P	0.2025	0	NA	NA	NA
**XRCC2**	P	0.00000423	0	0	0	NA
**RFWD3**	P	0.5253	0	0	0	NA

Additional Table 3. Comparison of Inflammation-related circuit activities between human pancreatic islets with Type 2 diabetes and normal ones. The last column shows the circuit functionalities according Uniprot.

Circuit	Log Fold Change	t	P-value	FDR-adj. p-value	Function (Uniprot)
**Rap1 signaling pathway: PRKCI PARD6A PARD3**	0.001003	3.835813	0.000297	0.008921	Inflammatory response, Cell cycle, Cell division, Differentiation
**NF-kappa B signaling pathway: CCL19**	0.01209	3.374508	0.001285	0.016761	Inflammatory response. Chemotaxis
**NF-kappa B signaling pathway: CCL21**	0.02025	3.287014	0.001676	0.016761	Inflammatory response. Chemotaxis
**NF-kappa B signaling pathway: CCL13**	0.007066	2.71083	0.008687	0.065155	Inflammatory response. Chemotaxis
**NF-kappa B signaling pathway: CXCL8**	0.004867	2.12408	0.037692	0.18395	Inflammatory response. Chemotaxis
**PPAR signaling pathway: OLR1**	0.00917	2.117447	0.038273	0.18395	Inflammatory response. Cell adhesion. Immunity
**Ras signaling pathway: PIK3R5***	0.00924	2.067291	0.042922	0.18395	Angiogenesis. Endocytosis. Autophagy. Cell adhesion. Chemotaxis. Adaptive immunity. Inflammatory response. Innate immunity. Differentiation. Immunity. Stress response. Host-virus interaction. Protein transport. Transport
**NF-kappa B signaling pathway: IL1B***	0.002005	1.833731	0.071533	0.268251	Inflammatory response. Pyrogen
**NF-kappa B signaling pathway: TNFAIP3**	0.002549	1.585311	0.118025	0.393415	Inflammatory response. Apoptosis.Ubl conjugation pathway
**Toll-like receptor signaling pathway: CXCL8**	0.005751	1.136375	0.260208	0.780625	Chemotaxis. Inflammatory response
**Toll-like receptor signaling pathway: CCL4L1**	-0.00104	-0.88544	0.37937	0.872459	Chemotaxis. Inflammatory response
**Toll-like receptor signaling pathway: IL1B**	0.002036	0.858897	0.393733	0.872459	Inflammatory response. Pyrogen
**NOD-like receptor signaling pathway: NLRP3 PYCARD**	-0.0003	-0.84108	0.403562	0.872459	Apoptosis. Inflammatory response. Innate immunity. Immunity
**Complement and coagulation cascades: C4A**	-0.00259	-0.73026	0.468006	0.872459	Complement pathway. Innate immunity. Inflammatory response. Immunity
**Complement and coagulation cascades: C3**	0.022158	0.727085	0.469933	0.872459	Complement pathway. Innate immunity. Inflammatory response. Complement alternate pathway. Fatty acid

					metabolism. Immunity. Lipid metabolism
**Toll-like receptor signaling pathway: CXCL10**	0.000774	0.712389	0.478918	0.872459	Chemotaxis. Inflammatory response
**NF-kappa B signaling pathway: CXCL2**	0.001214	0.603292	0.548533	0.872459	Inflammatory response. Chemotaxis
**NF-kappa B signaling pathway: CCL4L1**	0.000327	0.600263	0.550537	0.872459	Inflammatory response. Chemotaxis
**NOD-like receptor signaling pathway: PYCARD CASP1**	-0.00055	-0.56603	0.573434	0.872459	Apoptosis. Inflammatory response. Innate immunity. Immunity
**Chemokine signaling pathway: PARD3 PRKCZ TIAM1**	0.000103	0.540267	0.590964	0.872459	Cell cycle. Cell division. Differentiation. Inflammatory response
**NOD-like receptor signaling pathway: NLRC4 PYCARD**	-0.00061	-0.47478	0.636626	0.872459	Apoptosis. Inflammatory response. Innate immunity. Immunity
**Toll-like receptor signaling pathway: CXCL9**	0.000199	0.470302	0.639803	0.872459	Inflammatory response
**NOD-like receptor signaling pathway: CASP1 PYCARD**	-0.00095	-0.42411	0.672965	0.87778	Apoptosis. Inflammatory response. Innate immunity. Immunity
**Progesterone-mediated oocyte maturation: PIK3R5**	-0.00217	-0.33108	0.741706	0.916293	Angiogenesis. Endocytosis. Autophagy. Cell adhesion. Chemotaxis. Inflammatory response. Adaptive immunity. Innate immunity. Differentiation. Immunity. Stress response. Protein transport. Host-virus interaction. Transport
**Toll-like receptor signaling pathway: CCL3L3**	-0.00044	-0.29644	0.767893	0.916293	Chemotaxis. Inflammatory response
**Toll-like receptor signaling pathway: CCL5**	0.000328	0.224689	0.822966	0.916293	Chemotaxis. Inflammatory response
**Toll-like receptor signaling pathway: CXCL11**	-0.00018	-0.2225	0.824663	0.916293	Chemotaxis. Inflammatory response
**NOD-like receptor signaling pathway: NLRP3 CARD8 CASP1**	-0.0005	-0.12203	0.90327	0.967789	Apoptosis. Inflammatory response. Innate immunity. Immunity
**FoxO signaling pathway: BCL6**	0	3.37E-07	1	1	Inflammatory response. Transcription regulation. Transcription. Immunity
**FoxO signaling pathway: BCL6***	0	3.37E-07	1	1	Inflammatory response. Transcription regulation. Transcription. Immunity

Additional Table 4. Signaling circuits differentially activated in the comparison of human islets gene expression of healthy donors with type 2 diabetic patients. To limit the size of the table only 183 circuits, with a significant differential activity at a nominal p-value < 0.05, are listed.

CIRCUIT	UP / DOWN	statistic	p-value	FDR p-value	FunctionUniprot
**Adherens junction: NLK**	DOWN	-3,5159	0,0005	0,1469	Wnt signaling pathway, Transcription regulation, Transcription
**Adherens junction: IQGAP1****	DOWN	-3,4766	0,0005	0,1469	
**Wnt signaling pathway: MYC**	UP	3,3980	0,0007	0,1469	Transcription regulation, Transcription
**MAPK signaling pathway: MAPT**	DOWN	-3,3784	0,0008	0,1469	
**Rap1 signaling pathway: CDH1**	UP	3,3391	0,0009	0,1469	Cell cycle, Cell division, Cell adhesion, Mitosis, DNA repair, DNA damage, Ubl conjugation pathway
**Rap1 signaling pathway: PRKCI PARD6A PARD3**	UP	3,3195	0,0009	0,1469	Inflammatory response, Cell cycle, Cell division, Differentiation
**PI3K-Akt signaling pathway: MYC**	UP	3,2802	0,0011	0,1469	Transcription regulation, Transcription
**Retrograde endocannabinoid signaling: CACNA1A**	DOWN	-3,2802	0,0011	0,1469	Ion channel, Calcium channel, Calcium transport, Voltage-gated channel, Ion transport, Transport
**Retrograde endocannabinoid signaling: CACNA1A***	DOWN	-3,2802	0,0011	0,1469	Ion channel, Calcium channel, Calcium transport, Voltage-gated channel, Ion transport, Transport
**Thyroid hormone signaling pathway: PFKFB2**	DOWN	-3,2605	0,0012	0,1469	
**PI3K-Akt signaling pathway: BCL2L1***	UP	3,2409	0,0012	0,1469	Apoptosis, Endocytosis
**Toll-like receptor signaling pathway: CD86**	UP	3,2409	0,0012	0,1469	Host-virus interaction, Adaptive immunity, Immunity
**AMPK signaling pathway: PFKFB1**	DOWN	-3,1820	0,0015	0,1469	
**Retrograde endocannabinoid signaling: PRKACA**	DOWN	-3,1427	0,0017	0,1469	Apoptosis, Angiogenesis, Cell adhesion
**Rap1 signaling pathway: ACTB**	UP	3,1034	0,0020	0,1538	
**Rap1 signaling pathway: AKT3**	UP	3,0838	0,0021	0,1578	Glycogen biosynthesis, Glycogen metabolism, Glucose metabolism, Apoptosis, Sugar transport, Carbohydrate metabolism, Translation regulation, Neurogenesis,

					Transport
**PPAR signaling pathway: CPT2**	DOWN	-3,0248	0,0026	0,1651	Fatty acid metabolism, Lipid metabolism, Transport
**PI3K-Akt signaling pathway: CCND1**	UP	3,0248	0,0026	0,1651	Cell cycle, Cell division, DNA damage, Transcription regulation, Transcription
**PI3K-Akt signaling pathway: G6PC**	DOWN	-3,0052	0,0027	0,1651	Gluconeogenesis
**Rap1 signaling pathway: ITGAL**	UP	2,9659	0,0031	0,1818	Cell adhesion, Host-virus interaction
**Rap1 signaling pathway: MAPK14**	UP	2,9266	0,0035	0,1942	Stress response, Apoptosis, Transcription regulation, Transcription, Cell cycle
**Regulation of actin cytoskeleton: PFN3 ACTB**	DOWN	-2,9266	0,0035	0,1942	
**AMPK signaling pathway: SCD**	DOWN	-2,9070	0,0038	0,1953	Fatty acid metabolism, Fatty acid biosynthesis, Lipid metabolism, Lipid biosynthesis
**Gap junction: GJA1 GJA1*** **	UP	2,9070	0,0038	0,1953	
**PPAR signaling pathway: SCD**	DOWN	-2,8481	0,0045	0,2117	Fatty acid metabolism, Lipid metabolism, Fatty acid biosynthesis, Lipid biosynthesis
**PPAR signaling pathway: SCP2**	DOWN	-2,8481	0,0045	0,2117	Lipid transport, Meiosis, Cell division, Transport, Cell cycle
**Estrogen signaling pathway: AKT3***	UP	2,8481	0,0045	0,2117	Glycogen biosynthesis, Glycogen metabolism, Glucose metabolism, Sugar transport, Carbohydrate metabolism, Translation regulation, Apoptosis, Neurogenesis, Transport
**Thyroid hormone signaling pathway: TBC1D4**	DOWN	-2,8481	0,0045	0,2117	
**Wnt signaling pathway: CCND1**	UP	2,8284	0,0048	0,2178	Cell division, Cell cycle, Transcription regulation, Transcription, DNA damage
**MAPK signaling pathway: STK3**	UP	2,7891	0,0054	0,2178	Apoptosis
**PPAR signaling pathway: ACADM**	DOWN	-2,7499	0,0061	0,2178	Fatty acid metabolism, Lipid metabolism
**Adherens junction: ACTB VCL**	UP	2,7499	0,0061	0,2178	Cell adhesion
**Regulation of actin cytoskeleton: MYL12B MYH9 ACTB**	DOWN	-2,7302	0,0065	0,2178	Cell shape, Cell adhesion
**cGMP-PKG signaling pathway: PDE3A**	UP	2,7106	0,0069	0,2178	Angiogenesis
**Regulation of actin cytoskeleton: CFL1 ACTB**	DOWN	-2,7106	0,0069	0,2178	

**Retrograde endocannabinoid signaling: MAPK14***	UP	2,6909	0,0073	0,2178	Stress response, Apoptosis, Transcription regulation, Transcription, Cell cycle, Host-virus interaction, Biological rhythms
**Retrograde endocannabinoid signaling: MAPK14**	UP	2,6909	0,0073	0,2178	Stress response, Apoptosis, Transcription regulation, Transcription, Cell cycle, Host-virus interaction, Biological rhythms
**Regulation of actin cytoskeleton: ACTN4**	DOWN	-2,6909	0,0073	0,2178	Protein transport, Transport
**Insulin secretion: INS**	DOWN	-2,6909	0,0073	0,2178	Glucose metabolism, Carbohydrate metabolism
**Jak-STAT signaling pathway: MYC**	UP	2,6713	0,0078	0,2178	Transcription regulation, Transcription
**Fc epsilon RI signaling pathway: MAPK14**	UP	2,6713	0,0078	0,2178	Stress response, Apoptosis, Transcription regulation, Transcription, Cell cycle
**cAMP signaling pathway: AMH**	UP	2,6517	0,0082	0,2178	Gonadal differentiation, Differentiation
**Prolactin signaling pathway: CCND1****	UP	2,6517	0,0082	0,2178	Cell division, Cell cycle, Transcription regulation, DNA damage, Transcription
**PPAR signaling pathway: GK**	UP	2,6320	0,0087	0,2178	Glycerol metabolism
**Wnt signaling pathway: MMP7**	UP	2,6320	0,0087	0,2178	Collagen degradation
**Complement and coagulation cascades: CPB2**	UP	2,6320	0,0087	0,2178	Blood coagulation, Hemostasis, Fibrinolysis
**Toll-like receptor signaling pathway: NFKB1**	UP	2,6320	0,0087	0,2178	Apoptosis, Transcription regulation, Transcription
**Cytosolic DNA-sensing pathway: NFKB1***	UP	2,6320	0,0087	0,2178	Apoptosis, Transcription regulation, Transcription, Host-virus interaction
**Regulation of actin cytoskeleton: IQGAP2**	UP	2,6320	0,0087	0,2178	
**Estrogen signaling pathway: ESR1 C00951**	UP	2,6320	0,0087	0,2178	Transcription regulation, Transcription
**Estrogen signaling pathway: ESR1 FOS**	UP	2,6320	0,0087	0,2178	Transcription regulation, Transcription
**Estrogen signaling pathway: ESR1 C00951***	UP	2,6320	0,0087	0,2178	Transcription regulation, Transcription
**Wnt signaling pathway: PPARD**	UP	2,6124	0,0093	0,2178	Transcription regulation, Transcription
**Hippo signaling pathway: AREG**	UP	2,6124	0,0093	0,2178	
**Hippo signaling pathway: SERPINE1**	UP	2,6124	0,0093	0,2178	
**Hippo signaling pathway: CTGF***	UP	2,5927	0,0098	0,2178	DNA synthesis, Cell adhesion
**Progesterone-mediated oocyte maturation: ANAPC10**	UP	2,5927	0,0098	0,2178	Mitosis, Cell division, Cell cycle, Ubl conjugation pathway, Neurogenesis, Differentiation

**Estrogen signaling pathway: CREB3*** **	UP	2,5927	0,0098	0,2178	Unfolded protein response, Transcription regulation, Transcription, Host-virus interaction, Chemotaxis, Biological rhythms, Differentiation, DNA damage
**Thyroid hormone signaling pathway: WNT4**	DOWN	-2,5927	0,0098	0,2178	Wnt signaling pathway
**AMPK signaling pathway: CCND1**	UP	2,5731	0,0104	0,2200	Cell division, Cell cycle, DNA damage, Transcription regulation, Transcription
**Jak-STAT signaling pathway: CDKN1A**	UP	2,5731	0,0104	0,2200	Cell cycle
**Wnt signaling pathway: FOSL1**	UP	2,5534	0,0110	0,2276	
**Regulation of actin cytoskeleton: MSN**	DOWN	-2,5534	0,0110	0,2276	Cell shape
**Signaling pathways regulating pluripotency of stem cells: MYC***	UP	2,5338	0,0116	0,2381	Transcription regulation, Transcription
**Adherens junction: SNAI1**	UP	2,4945	0,0130	0,2417	
**Regulation of actin cytoskeleton: VCL***	DOWN	-2,4945	0,0130	0,2417	Cell adhesion
**Ras signaling pathway: RAC1****	UP	2,4749	0,0137	0,2417	
**HIF-1 signaling pathway: PFKFB3**	UP	2,4749	0,0137	0,2417	
**Hedgehog signaling pathway: PTCH1****	DOWN	-2,4749	0,0137	0,2417	
**Fc gamma R-mediated phagocytosis: PLA2G4B**	UP	2,4749	0,0137	0,2417	Lipid degradation, Lipid metabolism
**NF-kappa B signaling pathway: PLCG1**	DOWN	-2,4552	0,0145	0,2417	Host-virus interaction, Lipid degradation, Lipid metabolism
**Focal adhesion: VASP ACTB**	UP	2,4552	0,0145	0,2417	
**Prolactin signaling pathway: TNFSF11**	UP	2,4552	0,0145	0,2417	
**Oxytocin signaling pathway: MYL9 ACTB**	DOWN	-2,4552	0,0145	0,2417	
**Axon guidance: UNC5D PTPN11**	DOWN	-2,4356	0,0153	0,2417	Apoptosis
**Hippo signaling pathway: YWHAQ YAP1**	UP	2,4356	0,0153	0,2417	Host-virus interaction, Transcription regulation, Transcription
**Hippo signaling pathway: SMAD7**	UP	2,4356	0,0153	0,2417	Transcription regulation, Transcription
**Retrograde endocannabinoid signaling: ADCY1**	DOWN	-2,4356	0,0153	0,2417	cAMP biosynthesis, Biological rhythms, Olfaction, Sensory transduction
**Insulin secretion: CACNA1C**	DOWN	-2,4356	0,0153	0,2417	Calcium channel, Calcium transport, Voltage-gated channel, Ion channel, Ion transport, Transport, Vision,

					Sensory transduction
**Insulin secretion: CAMK2A**	DOWN	-2,4356	0,0153	0,2417	Neurogenesis, Differentiation
**Estrogen signaling pathway: CREB3**	UP	2,4356	0,0153	0,2417	Unfolded protein response, Transcription regulation, Transcription, Host-virus interaction, Chemotaxis, Biological rhythms, Differentiation, DNA damage
**Glutamatergic synapse: GRM3**	DOWN	-2,4258	0,0157	0,2462	
**PPAR signaling pathway: MMP1**	UP	2,4159	0,0161	0,2470	Collagen degradation, Host-virus interaction
**NF-kappa B signaling pathway: PLAU**	UP	2,3963	0,0170	0,2470	Fibrinolysis, Plasminogen activation, Blood coagulation, Hemostasis
**Regulation of actin cytoskeleton: ACTB ARPC5**	UP	2,3963	0,0170	0,2470	
**Regulation of actin cytoskeleton: GSN**	DOWN	-2,3963	0,0170	0,2470	Cilium biogenesis/degradation
**Insulin secretion: RAB3A**	DOWN	-2,3963	0,0170	0,2470	Exocytosis, Transport, Protein transport
**Axon guidance: ROCK1***	UP	2,3767	0,0179	0,2470	Apoptosis, Biological rhythms
**TNF signaling pathway: CEBPB**	UP	2,3767	0,0179	0,2470	Transcription regulation, Transcription, Differentiation
**Thyroid hormone signaling pathway: NOTCH1**	DOWN	-2,3767	0,0179	0,2470	Notch signaling pathway, Transcription regulation, Transcription, Differentiation, Angiogenesis
**Aldosterone-regulated sodium reabsorption: ATP1B4**	DOWN	-2,3767	0,0179	0,2470	Ion transport, Transport, Transcription regulation, Transcription, Sodium/potassium transport, Potassium transport, Sodium transport, Cell adhesion
**PPAR signaling pathway: FADS2**	DOWN	-2,3570	0,0189	0,2470	Fatty acid metabolism, Lipid metabolism, Fatty acid biosynthesis, Lipid biosynthesis, Transport, Electron transport
**Rap1 signaling pathway: RALA**	DOWN	-2,3570	0,0189	0,2470	Cell cycle, Cell division, Host-virus interaction, Exocytosis, Apoptosis
**MAPK signaling pathway: ECSIT TRAF6**	UP	2,3374	0,0199	0,2515	Innate immunity, Immunity, Osteogenesis, Ubl conjugation pathway
**Axon guidance: UNC5D DCC**	DOWN	-2,3374	0,0199	0,2515	Apoptosis
**HIF-1 signaling pathway: HMOX1**	UP	2,2981	0,0221	0,2597	Apoptosis
**Focal adhesion: BAD**	DOWN	-2,2981	0,0221	0,2597	Apoptosis
**Jak-STAT signaling pathway: MCL1**	UP	2,2981	0,0221	0,2597	Apoptosis, Differentiation

**PI3K-Akt signaling pathway: BCL2***	UP	2,2785	0,0233	0,2597	Apoptosis
**Antigen processing and presentation: CD4 HLA-DMA**	UP	2,2785	0,0233	0,2597	Immunity, Adaptive immunity, Host-virus interaction, MHC II, MHC I
**Fc gamma R-mediated phagocytosis: ARPC5**	UP	2,2785	0,0233	0,2597	
**Inflammatory mediator regulation of TRP channels: TRPV2**	UP	2,2785	0,0233	0,2597	Ion channel, Ion transport, Calcium transport, Calcium channel, Transport
**GnRH signaling pathway: JUN**	UP	2,2785	0,0233	0,2597	Transcription regulation, Transcription
**PPAR signaling pathway: DBI**	DOWN	-2,2588	0,0245	0,2597	Transport
**Ras signaling pathway: STK4 STK4**	UP	2,2588	0,0245	0,2597	Apoptosis
**HIF-1 signaling pathway: LDHA**	UP	2,2588	0,0245	0,2597	
**Thyroid hormone signaling pathway: CTNNB1**	UP	2,2588	0,0245	0,2597	Transcription regulation, Transcription, Wnt signaling pathway, Cell adhesion, Neurogenesis
**NF-kappa B signaling pathway: CCL19**	UP	2,2392	0,0258	0,2597	Inflammatory response, Chemotaxis
**Sphingolipid signaling pathway: PRKCA**	DOWN	-2,2392	0,0258	0,2597	Apoptosis, Angiogenesis, Cell adhesion, Transcription regulation, Transcription, Adaptive immunity, Immunity, Biological rhythms
**PI3K-Akt signaling pathway: GYS1**	UP	2,2392	0,0258	0,2597	Glycogen biosynthesis
**Jak-STAT signaling pathway: BCL2L1**	UP	2,2392	0,0258	0,2597	Apoptosis, Endocytosis
**cGMP-PKG signaling pathway: PDE2A**	DOWN	-2,2195	0,0271	0,2597	
**Hippo signaling pathway: SOX2**	DOWN	-2,2195	0,0271	0,2597	Transcription regulation, Transcription
**Adherens junction: ACTB**	UP	2,2195	0,0271	0,2597	
**Tight junction: IGSF5 TJP1 MLLT4**	UP	2,2195	0,0271	0,2597	Host-virus interaction, Cell adhesion, Angiogenesis
**RIG-I-like receptor signaling pathway: NFKB1**	UP	2,2195	0,0271	0,2597	Transcription regulation, Transcription, Apoptosis, Host-virus interaction
**Jak-STAT signaling pathway: GFAP**	UP	2,2195	0,0271	0,2597	
**Estrogen signaling pathway: CREB3***	DOWN	-2,2195	0,0271	0,2597	Unfolded protein response, Transcription regulation, Transcription, Host-virus interaction, Chemotaxis, Biological rhythms, Differentiation, DNA damage
**Oxytocin signaling pathway: CDKN1A**	UP	2,2195	0,0271	0,2597	Cell cycle

**Aldosterone synthesis and secretion: ITPR1**	DOWN	-2,2195	0,0271	0,2597	Ion channel, Calcium channel, Ligand-gated ion channel, Calcium transport, Ion transport, Transport, Apoptosis
**Axon guidance: NFATC2**	DOWN	-2,1999	0,0285	0,2649	Transcription regulation, Transcription, Differentiation
**Dopaminergic synapse: CAMK2A**	DOWN	-2,1999	0,0285	0,2649	Neurogenesis, Differentiation
**Glucagon signaling pathway: GYS1**	UP	2,1999	0,0285	0,2649	Glycogen biosynthesis
**Adherens junction: CTNND1**	DOWN	-2,1802	0,0300	0,2649	Wnt signaling pathway, Cell adhesion, Transcription regulation, Transcription
**Jak-STAT signaling pathway: BCL2**	UP	2,1802	0,0300	0,2649	Apoptosis
**Natural killer cell mediated cytotoxicity: KLRC4-KLRK1**	UP	2,1802	0,0300	0,2649	Adaptive immunity, Innate immunity, Differentiation, Immunity
**GABAergic synapse: CACNA1A***	DOWN	-2,1802	0,0300	0,2649	Ion channel, Ion transport, Calcium channel, Calcium transport, Voltage-gated channel, Transport, Vision, Sensory transduction
**Regulation of actin cytoskeleton: ARPC5 ACTB**	UP	2,1802	0,0300	0,2649	
**Regulation of actin cytoskeleton: ARPC5 ACTB***	UP	2,1802	0,0300	0,2649	
**Insulin secretion: PRKCA**	DOWN	-2,1802	0,0300	0,2649	Angiogenesis, Apoptosis, Cell adhesion, Adaptive immunity, Immunity, Transcription regulation, Transcription, Biological rhythms
**Ras signaling pathway: PIK3R5***	UP	2,1606	0,0315	0,2649	Angiogenesis, Endocytosis, Autophagy, Cell adhesion, Chemotaxis, Adaptive immunity, Inflammatory response, Innate immunity, Differentiation, Immunity, Stress response, Host-virus interaction, Protein transport, Transport
**Rap1 signaling pathway: THBS1**	UP	2,1606	0,0315	0,2649	Cell adhesion, Unfolded protein response
**Tight junction: TJP1 YBX3 CDK4**	UP	2,1606	0,0315	0,2649	Transcription regulation, Transcription, Cell division, Cell cycle
**Jak-STAT signaling pathway: CCND1**	UP	2,1606	0,0315	0,2649	Cell cycle, Cell division, DNA damage, Transcription regulation, Transcription
**T cell receptor signaling pathway: CD3E**	UP	2,1606	0,0315	0,2649	
**B cell receptor signaling pathway: RAC1**	UP	2,1606	0,0315	0,2649	Transcription regulation, Transcription

**Adipocytokine signaling pathway: AGRP**	UP	2,1606	0,0315	0,2649	
**Adipocytokine signaling pathway: POMC**	DOWN	-2,1606	0,0315	0,2649	
**NF-kappa B signaling pathway: CXCL12**	UP	2,1410	0,0331	0,2673	Chemotaxis
**PI3K-Akt signaling pathway: BCL2L1**	UP	2,1410	0,0331	0,2673	Apoptosis, Endocytosis
**Toll-like receptor signaling pathway: CASP8**	UP	2,1410	0,0331	0,2673	Host-virus interaction, Apoptosis
**Natural killer cell mediated cytotoxicity: TNFRSF10D**	UP	2,1410	0,0331	0,2673	Apoptosis
**NF-kappa B signaling pathway: TNFSF13B***	UP	2,1213	0,0347	0,2679	Immunity
**HIF-1 signaling pathway: EDN1**	UP	2,1213	0,0347	0,2679	
**Apoptosis: TNFRSF10D***	UP	2,1213	0,0347	0,2679	Apoptosis
**Wnt signaling pathway: JUN**	UP	2,1213	0,0347	0,2679	Transcription regulation, Transcription
**TNF signaling pathway: BAG4**	UP	2,1213	0,0347	0,2679	
**NF-kappa B signaling pathway: CCL13**	UP	2,1017	0,0365	0,2679	Inflammatory response, Chemotaxis
**Adipocytokine signaling pathway: PTPN11**	DOWN	-2,1017	0,0365	0,2679	
**MAPK signaling pathway: JUND**	DOWN	-2,0820	0,0382	0,2715	Transcription regulation, Transcription
**ErbB signaling pathway: MYC**	UP	2,0820	0,0382	0,2715	Transcription regulation, Transcription
**Ras signaling pathway: NFKB1**	UP	2,0820	0,0382	0,2715	Apoptosis, Transcription regulation, Transcription, Host-virus interaction
**Sphingolipid signaling pathway: RAC1**	UP	2,0820	0,0382	0,2715	Transcription regulation, Transcription
**Complement and coagulation cascades: F2RL2**	UP	2,0820	0,0382	0,2715	Blood coagulation, Hemostasis
**Endocrine and other factor-regulated calcium reabsorption: TRPV5***	UP	2,0820	0,0382	0,2715	Calcium transport, Ion transport, Calcium channel, Transport, Ion channel
**cAMP signaling pathway: CACNA1C**	DOWN	-2,0624	0,0401	0,2746	Ion transport, Ion channel, Calcium transport, Calcium channel, Voltage-gated channel, Transport, Vision, Sensory transduction
**Melanogenesis: TYRP1**	UP	2,0624	0,0401	0,2746	Melanin biosynthesis
**Sphingolipid signaling pathway: SMPD1**	DOWN	-2,0428	0,0421	0,2746	
**Axon guidance: GSK3B**	DOWN	-2,0428	0,0421	0,2746	Neurogenesis, Differentiation, Glycogen metabolism, Biological rhythms, Carbohydrate metabolism, Wnt signaling pathway

**Tight junction: OCLN OCLN**	UP	2,0428	0,0421	0,2746	
**Signaling pathways regulating pluripotency of stem cells: AKT3***	UP	2,0428	0,0421	0,2746	Glycogen biosynthesis, Glycogen metabolism, Glucose metabolism, Sugar transport, Carbohydrate metabolism, Apoptosis, Translation regulation, Neurogenesis, Transport
**Circadian entrainment: PRKCA**	DOWN	-2,0428	0,0421	0,2746	Angiogenesis, Apoptosis, Cell adhesion, Adaptive immunity, Transcription regulation, Transcription, Immunity, Biological rhythms
**Insulin signaling pathway: G6PC**	DOWN	-2,0428	0,0421	0,2746	Gluconeogenesis
**Melanogenesis: CAMK2A**	UP	2,0428	0,0421	0,2746	Neurogenesis, Differentiation
**Thyroid hormone signaling pathway: MYC**	UP	2,0428	0,0421	0,2746	Transcription regulation, Transcription
**PPAR signaling pathway: EHHADH**	DOWN	-2,0231	0,0441	0,2746	Fatty acid metabolism, Lipid metabolism
**HIF-1 signaling pathway: PDHA1**	UP	2,0231	0,0441	0,2746	Glucose metabolism, Tricarboxylic acid cycle, Carbohydrate metabolism
**Apoptosis: PMAIP1**	UP	2,0231	0,0441	0,2746	Apoptosis
**Leukocyte transendothelial migration: RAC2**	UP	2,0231	0,0441	0,2746	
**Aldosterone synthesis and secretion: KCNK3**	UP	2,0231	0,0441	0,2746	Ion channel, Ion transport, Potassium channel, Potassium transport, Voltage-gated channel, Transport
**Adherens junction: CDH1***	UP	2,0035	0,0462	0,2746	Cell adhesion, Cell division, Cell cycle, Mitosis, DNA repair, DNA damage, Ubl conjugation pathway
**PPAR signaling pathway: ME1**	DOWN	-2,0035	0,0462	0,2746	
**MAPK signaling pathway: STMN1**	DOWN	-2,0035	0,0462	0,2746	Neurogenesis, Differentiation
**Rap1 signaling pathway: RAC1**	DOWN	-2,0035	0,0462	0,2746	Transcription regulation, Transcription
**FoxO signaling pathway: IL7R**	UP	2,0035	0,0462	0,2746	
**Hedgehog signaling pathway: SMO**	UP	2,0035	0,0462	0,2746	
**Cytosolic DNA-sensing pathway: NFKB1**	UP	2,0035	0,0462	0,2746	Apoptosis, Transcription regulation, Transcription, Host-virus interaction
**TNF signaling pathway: MAPK14***	UP	2,0035	0,0462	0,2746	Transcription regulation, Transcription, Stress response, Apoptosis, Cell cycle
**Serotonergic synapse: CACNA1C**	DOWN	-2,0035	0,0462	0,2746	Ion channel, Calcium channel, Ion transport, Calcium

					transport, Voltage-gated channel, Transport, Vision, Sensory transduction
**Oxytocin signaling pathway: C00219**	UP	2,0035	0,0462	0,2746	
**HIF-1 signaling pathway: TFRC**	UP	1,9838	0,0484	0,2823	Endocytosis, Host-virus interaction
**p53 signaling pathway: PMAIP1**	UP	1,9838	0,0484	0,2823	Apoptosis
**Signaling pathways regulating pluripotency of stem cells: MAPK1***	UP	1,9838	0,0484	0,2823	Transcription regulation, Transcription, Apoptosis, Cell cycle, Host-virus interaction

Additional Figure 1. Correlation between LoF simulation effects on the circuits and the population frequency of LoF mutations. LoF mutations were simulated in all the genes of circuits (from left to right) Rap1 signaling pathway:PRKCI-PARD6A-PARD3, Chemokynes pathway:PARD3-PRKCZ-TIAM1 and TNF signaling pathway:CREB3 and the -log(p-value) of the differential activity caused in the corresponding circuit is plotted against the population frequency at which this gene appear in the healthy population (1000 genomes) with a LoF mutation.

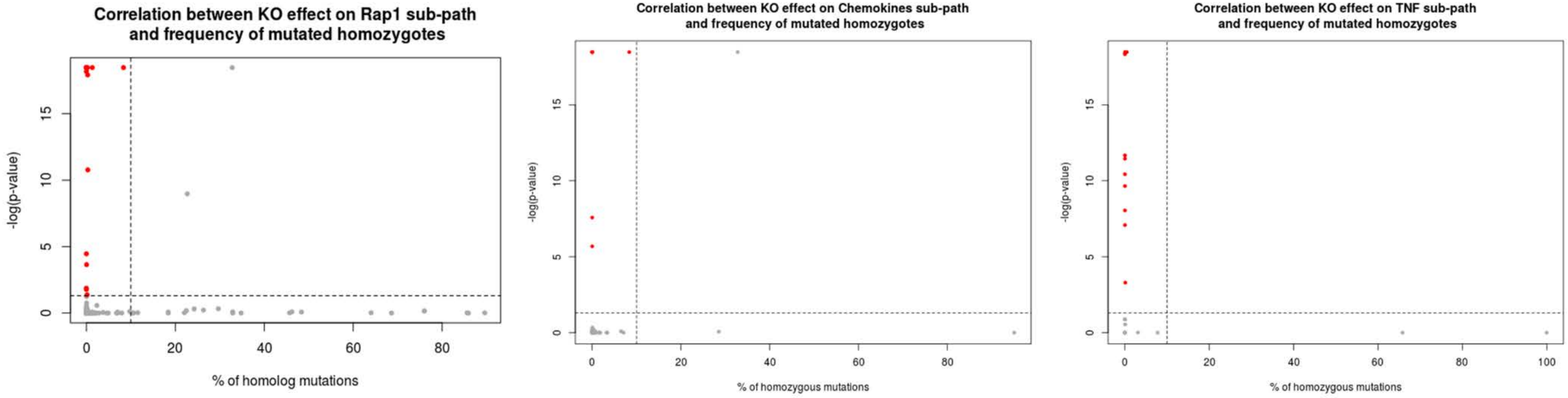

Supplement: Supplementary file 1 — Supplementary information [file 41598_2019_55454_MOESM1_ESM.pdf]
